# Comparative Assessment of the Kinetics of Cellular and Humoral Immune Responses to COVID-19 Vaccination in Cancer Patients

**DOI:** 10.3390/v15071439

**Published:** 2023-06-26

**Authors:** Lina Souan, Hikmat Abdel-Razeq, Muna Al Zughbieh, Sara Al Badr, Maher A. Sughayer

**Affiliations:** 1 Laboratory Medicine, Department of Pathology, King Hussein Cancer Center, Amman 11941, Jordan; 2 Department of Medicine, King Hussein Cancer Center, Amman 11941, Jordan

**Keywords:** BNT162b2, BBIBP-CorV-virus inactivated vaccine, IFN-γ, T-cell response, IgG antibody titer

## Abstract

Objective: The kinetics of immune responses to various SARS-CoV-2 vaccines in cancer patients were investigated. Methods: In total, 57 cancer patients who received BNT162b2-RNA or BBIBP-CorV vaccines were enrolled. Cellular and humoral immunity were assessed at three-time points, before the first vaccine dose and 14–21 days after the first and second doses. Chemiluminescent microparticle immunoassay was used to evaluate SARS-CoV-2 anti-spike IgG response, and QuantiFERON^®^ SARS-CoV-2 kit assessed T-cell response. Results: Data showed that cancer patients’ CD4^+^ and CD8^+^ T cell-median IFN-γ secretion of SARS-CoV-2 antigens increased after the first and second vaccine doses (*p* = 0.027 and *p* = 0.042). BNT162b2 vaccinees had significantly higher IFN-γ levels to CD4^+^ and CD8^+^ T cell epitopes than BBIBP-CorV vaccinees (*p* = 0.028). There was a positive correlation between IgG antibody titer and T cell response regardless of vaccine type (*p* < 0.05). Conclusions: This study is one of the first to investigate cellular and humoral immune responses to SARS-CoV-2 immunization in cancer patients on active therapy after each vaccine dose. COVID-19 immunizations helped cancer patients develop an effective immune response. Understanding the cellular and humoral immune response to COVID-19 in cancer patients undergoing active treatment is necessary to improve vaccines and avoid future SARS pandemics.

## 1. Introduction

The COVID-19 pandemic has prompted unprecedented global challenges. Since its emergence in December 2019, the virus has infected millions of individuals, causing severe respiratory illness and a significant number of fatalities [1]. Global efforts were combined with the fight against SARS-CoV-2 to accelerate research to find vaccines against the newly emerging disease, a critical step in containing the pandemic [2,3,4,5]. Studies showed that SARS-CoV-infected and non-SARS-CoV-infected healthy individuals have T-cells reactive to SARS-CoV-2 proteins [6,7]. These findings indicate that various human coronaviruses, including common cold viruses, produce cross-reactive memory T-cells that react to the SARS-CoV-2 virus [6,8]. As for the humoral immune response, antibodies to the virus can also be detected in asymptomatic [9,10,11] and symptomatic individuals [12,13,14]. Longitudinal studies are being conducted to better understand the link between infection state, host immunity, and disease progression.

Developed vaccines must induce both humoral and cellular anti-SARS-CoV-2 immunity for protection against the SARS-CoV-2 virus [15,16,17]. Currently, several vaccines have been authorized for use by regulatory agencies, and they seem to be effective in preventing COVID-19 disease. A unique aspect of these vaccines is their ability to induce both humoral and cellular immunity, which plays a pivotal role in providing long-term protection against the virus. In addition, it is crucial to note that natural infection with the SARS-CoV-2 virus induces both humoral and cellular immunity. However, vaccines have a number of advantages over natural immunity, including the prevention of severe illness and hospitalization, a decrease in the risk of long-term complications, and the control of the virus’s dissemination among populations.

Studies showed that CD8^+^ T-cells are active responders to COVID-19 viral antigens and are critical cells of adaptive immunity [18,19]. Patients with SARS-CoV-2 infection who are asymptomatic or have a moderate illness produce strong virus-specific and long-lasting CD8^+^ T-cell responses. In mild SARS-CoV-2 infection, more interferon is produced than in severe cases [20,21].

Cancer patients undergoing active treatment may not respond adequately to the COVID-19 vaccine, particularly those with hematological malignancies. They may have a less effective immune response to COVID-19 vaccination than healthy people may. Studies showed that the efficiency of mRNA vaccinations in cancer patients against COVID-19 hospitalization is predicted to be around 75%, which is lower than the 90% in immunocompetent individuals [22]. Studies demonstrated that cancer patients have a seroconversion rate of 80% to 95% after the second dose of the SARS-CoV-2 vaccine [21,22]. In addition, research revealed that the specific type of cancer, its stage, and the patient’s clinical status [23] could influence the antibody response. For example, some studies showed no significant difference between cancer patients in the early stages who do not have metastases and normal individuals in terms of the severity of COVID-19 infection [24]. However, other studies reported that patients with lung cancer and hematological malignancies have lower antibody responses than those with solid tumors, which increases their susceptibility to COVID-19 infection [25,26,27]. Patients undergoing active cancer therapy are particularly vulnerable to COVID-19 infection because they have a higher risk of severe disease progression and mortality and are less likely to accept a vaccine. However, little is known about this population’s unique T-cell and humoral B-cell response [28,29]. In addition, immunocompromised patients should be evaluated regularly to check their immune status against SARS-CoV-2 and to determine a potential booster dose based on their active therapies [30,31]. Hence, the purpose of this study is to investigate the efficacy of BNT162b2-RNA (BioNTech, Pfizer-BioNTech, Mainz, Germany) and the BBIBP-CorV-virus inactivated (BBIBP-CorV BBIBP) vaccines in inducing both cellular and humoral immune responses to SARS-CoV-2 virus in adult cancer patients at King Hussein Cancer Center (KHCC).

## 2. Materials and Methods

### 2.1. Study Design

This prospective cohort study was conducted between November 2021 and July 2022 at KHCC, Jordan. In total, 57 adult cancer patients who received the COVID-19 vaccine were enrolled. All participants completed and signed a consent form approved by KHCC-Institutional Review Board (IRB). The Ethics Committee at the King Hussein Cancer Center approved the study (#21KHCC174F). Medical records for the study’s participants were reviewed for patients’ age, type of cancer, and cancer grade and treatment. Most participants received at least two doses of the COVID-19 vaccine, either the BNT162b2-RNA vaccine (Pfizer-BioNTech) or the BBIBP-CorV-virus inactivated (BBIBP-CorV-BBIBP-CorV) vaccine. The Jordanian Ministry of Health records were consulted for the history of COVID-19 infection and the type of vaccination.

### 2.2. Blood Sampling

Cellular and humoral immunity was assessed for each participant at three time intervals termed time point zero (T0). At this time point, the blood sample was drawn from the patient 1–7 days before the first vaccination dose. The second time point was called time point one (T1). This blood sample was drawn 14–21 days after the first vaccination. The third sample was collected from the patients 14–21 days after the second vaccination and labeled time point two (T2). Both whole blood and serum samples were collected at each time point to evaluate the cellular and humoral responses according to the test manufacturer’s instructions. Although the time interval between each vaccine dose and sampling was 14–21 days, the durations between immunization doses were not consistent. Regardless of vaccination type, the mean time interval between the first and second vaccine dose was 31.73 days, with a median of 23 days and a range of 18–118 days.

### 2.3. Quantitative IgG Antibodies Detection

In order to evaluate the humoral response, venous blood samples of five milliliters was taken at each time point from cancer patients. The separation of the serum was carried out by centrifuging the samples for 15 min at a relative centrifugal force (rcf) of 4300. After that, the samples were kept at a temperature of −80 degrees Celsius until the test was carried out. On the day of the test, the samples were progressively thawed by placing them in the refrigerator at 4 °C for 24 h, then placing them at room temperature for at least one hour before the test. Samples were tested using the SARS-CoV-2 IgG II Quant assay. This assay is a chemiluminescent microparticle immunoassay (CMIA) intended for the quantitative measurement of IgG antibodies to SARS-CoV-2, including neutralizing antibodies, to the receptor-binding domain (RBD) of the S1 subunit of the spike protein (Abbott Architect SARS-CoV-2 IgG). According to the manufacturer’s instructions, the test was performed on an Architect i1000 analyzer (Abbott, Chicago, IL, USA). The test quantification ranges from 21 to 40,000 AU/mL, with a positive result of ≥50 AU/mL and 100% favorable agreement with the plaque reduction neutralization tests (PRNT) [23]. The following conversion factor to calculate the World Health Organization (WHO) binding antibody units per mL (BAU/mL). BAU/mL is an arbitrary unit for comparing tests detecting the same type of immunoglobulins with the same specificity and the conversion equation used was used: 1 BAU/mL corresponds to 0.142 Abbott AU/mL [24].

### 2.4. Commercial IFN-γ Release Assay

Cellular immune response was assessed using the QuantiFERON^®^ SARS-CoV-2 RUO (QIAGEN) commercial assay. The test consists of a set of blood collection tubes which are coated by one of the following three antigens. The SARS-CoV-2 Ag1, SARS-CoV-2 Ag2, and SARS-CoV-2 Ag3 that use a combination of antigens specific to SARS-CoV-2 were used to stimulate lymphocytes in heparinized tubes for testing cell-mediated immunity (QuantiFERON SARS-CoV-2 Research Use Only; QIAGEN, Hilden, Germany). According to the manufacturer, the SARS-CoV-2 Ag1 tube contains CD4^+^ T-cell epitopes derived from the spike protein’s S1 subunit (receptor binding domain (RBD)) (Ag1). The Ag2 tube contains CD4^+^ and CD8^+^ T-cell epitopes from the S1 and S2 subunits of the S protein (Ag2), while the Ag3 tube consists of CD4^+^ and CD8^+^ epitopes from S1 and S2 plus immunodominant CD8^+^ epitopes derived from the whole SARS-CoV-2 genome (Ag3). Blood tubes containing nil and mitogen tests are used alongside these tubes as negative and positive controls. Specimens were processed per the manufacturer’s guidelines [25,26,27]. Plasma from the stimulated samples was used to detect IFN-γ using QuantiFERON ELISA. All tubes were incubated for 16–24 h at 37 °C, then centrifuged for 15 min at 2500× *g*. The supernatant was transferred to another tube and stored at −80 °C until analyzed. Frozen samples were thawed in the same manner as the serum samples. Following the manufacturer’s instructions, the IFN-γ level was measured using the enzyme-linked immunosorbent method (ELISA). Final IFN-γ values (IU/mL) for CD4^+^, CD4^+^, and CD8^+^, and mitogen were obtained by subtracting the nil value from the raw data.

### 2.5. Statistical Analysis

Data presentation and quantitative method comparison was performed using SPSS-IBM software version 28 (IBM SPSS Statistics, Chicago, IL, USA). Since the data were not normally distributed, non-parametric inferential statistics were used instead. The Friedman test examined the differences in the cellular immune response for each COVID-19 T-cell epitope at each time point. The Mann–Whitney U test was applied to compare T-cell response to COVID-19 antigens between the two vaccine types. The Spearman rank correlation was used to correlate the antibody titer with the three COVID-19 antigens; the *p*-value set at <0.05 is considered statistically significant.

## 3. Results

This study included 57 cancer patients. There were 44 (77.19%) females and 13 (22.81%) males with a mean age of 49.9 ± 11.6. Three-quarters of the participants had solid tumors, and 30 patients (52.63%) took the BNT162b2 COVID-19 vaccine, while 27 patients (47.37%) took the BBIBP-CorV vaccine (Table 1).

Among the participants, there were 23 patients (40.4%) who were on active chemotherapy, 10 (17.5%) patients on hormonal therapy, 1 patient (1.8%) on radiotherapy, 2 patients (3.5%) were on immunotherapy, and 21 patients (36.8%) did not receive any cancer treatment in the three months before the study or while it was being conducted (Table 2).

Data collected after the first vaccination dose reported 21 patients (36.8%) had no vaccine side effects, and 31 patients (54.4%) had mild side effects, such as chills, headache, and fatigue. While two patients (3.5%) had moderate side effects, which consisted of fever above 38°C and chills that needed medication to control, three patients (5.3%) experienced severe side effects like fever, necessitating hospitalization.

As for the reported vaccine side effects after the second dose, there were only 45 patients who took the second vaccine dose. Out of these participants, 19 patients (42.2%) did not report any side effects, 24 patients (53.3%) reported mild side effects, 1 patient (2.2%) had moderate side effects, and another (2.2%) reported severe side effects.

### 3.1. T-Cell Response to COVID-19 Antigens t Different Time Points for All Vaccine Types

**Figure 1:**
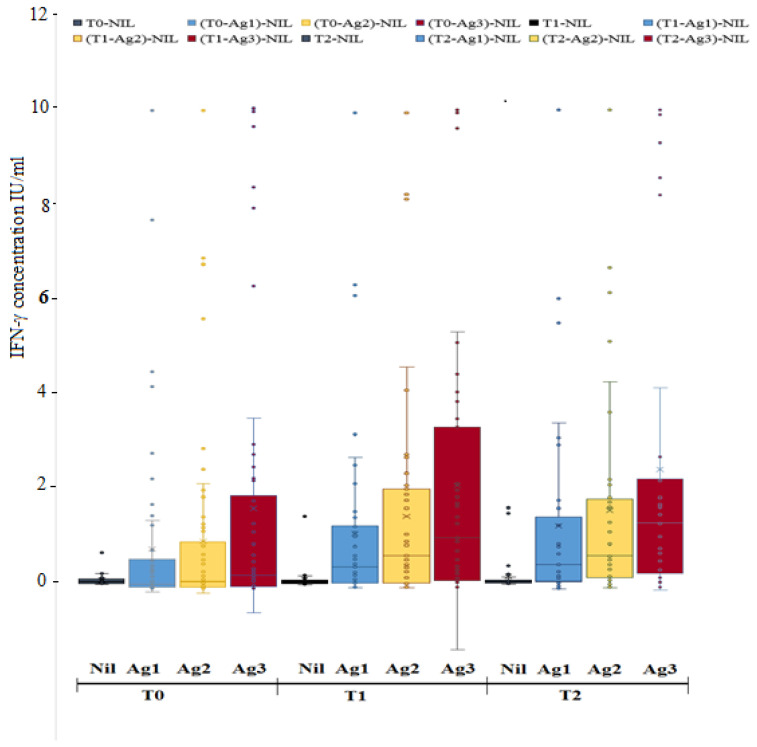
Comparison of the median differences between the three measurement times for the QFN SARS-CoV-2 antigen response.

The Friedman test examined the differences in the cellular immune response for each SARS-CoV-2 T-cell epitope at each time point. Data analysis revealed a statistically significant difference in IFN-γ secretion across the three time points when comparing the median differences of CD4^+^ T-cell responses to COVID-19 (Ag1) (X^2^ = 6.138, *p* = 0.046). A Bonferroni post hoc test showed that IFN-γ levels secreted at T0 had a significantly lower median compared to IFN-γ levels measured at T1 and T2 (*p* = 0.027 and *p* = 0.042), respectively, while there were no statistically significant median differences in IFN-γ levels between T1 and T2 (*p* = 0.854) (Figure 1). Likewise, the data showed that there was a statistically significant difference between the three time points concerning the median differences in IFN-γ levels that were assessed as a result of stimulation with COVID-19 (Ag2) (X^2^ = 9.937, *p* = 0.007). A Bonferroni post hoc test showed that IFN-γ levels measured at T0 had a significantly lower median than IFN-γ levels measured at both T1 and T2, *p* = 0.007 and *p* = 0.008, respectively. At the same time, there were no statistically significant median differences between T1 and T2, *p* = 0.951 (Figure 1). However, there was no statistically significant difference in the median IFN-γ levels at each of the three-time points (X^2^ = 4.299, *p* = 0.117) for COVID-19 (Ag3) (Figure 1).

### 3.2. Comparing T-Cell Response to COVID-19 Antigens According to Vaccine Types

**Figure 2:**
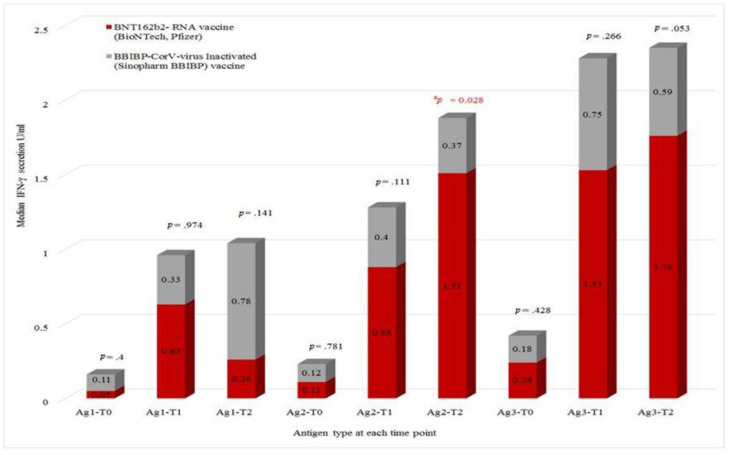
Comparative analysis of T-cell responses to COVID-19 antigens following immunization with either BNT162b2 or BBIBP-CorV. *: significant difference.

We compared the IFN-γ secretion in response to the three COVID-19 antigens across the three measurement times according to COVID-19 vaccine type (BNT162b2 vs. BBIBP-CorV). The data revealed that patients who received the BNT162b2 vaccine had significantly higher IFN-γ levels in response to Ag2 than those who received the BBIBP-CorV vaccine (*p* = 0.028). At the same time, there was no statistically significant median difference for the other measurement times when using the Mann–Whitney U test (Figure 2).

To determine whether there are any variations in the T-cell response to various antigens at various time points, the Wilcoxon signed ranks test was utilized for each type of immunization. The results showed a significant difference in response to Ag2 between T0-and T2 in the BNT162b2 vaccinated patient group (*p*-value = 0.036). While in the BBIBP-CorV vaccinated group, the significant difference in T-cell response was to Ag1 between T0 and T2 (*p*-value = 0.010). As for the remaining subgroups in the BNT162b2 and BBIBP-CorV vaccinated patients, there were some marginal differences, as seen in Figure 2, but they were not statistically significant.

### 3.3. Correlation between Neutralizing Antibody Response and Cellular Immune Response in Vaccinated Cancer Patients

We used the Friedman test for repeated measures to find whether the IgG antibody titer differs between the three-time points regardless of vaccine type. The data showed that there is a median difference between T0, T1, and T2 measured in BAU/mL units (T0 median = 121.51 BAU/mL, T1 median = 760.12 BAU/mL, T2 = 1335.37 BAU/mL) *p*-value < 0.001, respectively. While there were no significant median differences between T1 and T2 *p* value = 0.446. Moreover, we performed the Spearman rho test to investigate if there is a correlation between the antibody titer and IFN-γ secretion in response to each antigen at each time point regardless of the vaccine type. Table 3 revealed a statistically significant positive correlation between antibody titer and Ag1, Ag2, and Ag3 at both T1 and T2 time points.

## 4. Discussion

It is known that cellular immunity is essential in managing viral infections because of its effectiveness and correlation with better outcomes [28]. The pandemic of COVID-19 has prompted an unprecedented strive to create effective vaccines against the disease. As more individuals are immunized, there is an increasing urgency to understand the immediate and long-term effects of the vaccine on the immune system.

T-cells play an important role in the immune response to SARS-CoV-2 infection by limiting the severity of the disease [29], and our knowledge of their potential in vaccine-induced protection still needs to be investigated. The effect of the first and second dose of the COVID-19 vaccine on the activity of CD4^+^ and CD8^+^ T-cells is of particular interest. Studies showed that T-cells induced by either spontaneous infection [2,30,31] or by COVID-19 vaccination exhibited similar functional capabilities in healthy individuals [32], and the induced T-cell response was able to protect against the original SARS-CoV-2 and its variant’s infections [33]. Research has shown that the first dose of the COVID-19 vaccine can induce a strong immune response in vaccinated individuals. Specifically, studies have shown that the vaccine can stimulate the production of neutralizing antibodies [34], activate CD4^+^ T-cells, and generate a CD8^+^ T-cell response [35]. However, there is currently limited data on the effect of the second vaccine dose on CD4^+^ and CD8^+^ T-cell activity.

In cancer patients, current research suggests that cellular immunity is more robust than humoral immunity and may even be more predictive of SARS-CoV-2 protection in vaccinated cancer patients [36]. Cancer patients undergoing active treatment are more susceptible to severe COVID-19 infections and have poorer outcomes than non-cancer patients [37]. This study provides an integrated analysis of the efficacy of COVID-19 vaccines to induce cellular and humoral immune responses in cancer patients undergoing active cancer therapy following each vaccine dose.

Our data analysis showed that COVID-19 vaccines, regardless of the vaccine type, induced a significantly higher CD4^+^ and CD8^+^ T-cell response after the first and second vaccine dose compared to the baseline point before vaccination. This finding indicates that cancer patients were able to mount a cellular immune response to SARS-CoV-2 antigens, as previously reported [38]. The reported significant CD4^+^ and CD8^+^ T-cell response induced by the S1-RBD subunit (Ag1) and the S1 and S2 subunits of the S protein CD8^+^ T-cell epitopes (Ag2) after the first and second vaccine dose was similar to COVID-19 vaccinated healthy individuals reported in previous studies [39,40]. However, our data showed no statistically significant median differences in T-cell activation between the first and second vaccine doses, similar to earlier reports on healthy individuals [41,42]. Furthermore, our data showed that after the second dose, T-cells produced less IFN-γ in cancer patients similar to what was reported in healthy individuals [43], which may indicate a downregulation in the immune response by regulatory T-cells (Treg) similar to healthy individuals. Antiviral vaccination was shown to boost Treg, which limits excessive immune responses and prevents inflammatory damage, which might impair vaccine efficacy [44].

It is worth noting that T-cells in naive (not-vaccinated) cancer patients responded to all three COVID-19 antigens, possibly due to prior exposure to these antigens in a previous SARS-CoV-2 infection; however, this response was minimal in comparison to the cellular immune response induced after the first dose of vaccine.

Furthermore, we reported that T-cell activation increased following the first vaccination dose to CD4^+^ and CD8^+^ epitopes derived from S1 and S2 (Ag2) and immunodominant CD8^+^ epitopes derived from the entire SARS-CoV-2 genome (Ag3). However, this increase was not statistically significant. Nevertheless, this T-cell stimulation may demonstrate the development of a helper, cytotoxic, and memory T-cell response to immunodominant CD8^+^ epitopes generated from the SARS-CoV-2 genome, other than those on the spike protein.

Comparing the T-cell response generated by COVID-19 antigens between the two vaccine types, our data showed a twofold increase in T-cell response to S1-RBD-CD4^+^ T-cell epitopes (Ag1) in patients vaccinated with the BNT162b2 compared to those who were primed with BBIBP-CorV vaccine. Moreover, there was almost a 2.4-fold increase in T-cell activation to the S1 and S2 subunit-epitopes (Ag2) in patients vaccinated with BNT162b2 compared to patients primed and then boosted with the BBIBP-CorV vaccine, which is similar to previous reports in healthy individuals [45].

Most importantly, our data showed that the BNT162b2 vaccine induced a significantly higher CD4^+^ and CD8^+^ T-cell (Ag2) response after the second vaccination compared to the BBIBP-CorV vaccine in cancer patients. On the other hand, the BBIBP-CorV vaccine induced an almost three-fold increase in CD4^+^ T-cell response to S1-RBD subunit CD4^+^ epitopes (Ag1) after the second vaccine compared to the BNT162b2 vaccine.

Moreover, our results showed that there was a significant positive association between the IgG antibody titer level and CD4^+^ and CD8^+^ T-cell response to COVID-19 epitopes (Ag1), (Ag2), and (Ag3) after the first vaccine prime (T1) and the second vaccination (T2) time points similar to previous studies published on non-cancer individuals infected with SARS-CoV-2 virus [46,47,48]. A robust immune response against SARS-CoV-2 requires two phases, neutralization and effector T-cell functions, and the BNT162b2 vaccine effectively induces both humoral and cell-mediated immune responses in anti-PD 1/PDL-1-treated cancer patients. In addition, researchers demonstrated that the BNT162b2 vaccine was deemed safe and that these patients achieved satisfactory serologic status [49].

There were limitations in this study, such as the small sample size in each group. Yet, we could still statistically compare the various groups and identify essential variations. Nevertheless, additional research is required on larger populations of cancer patients undergoing active cancer treatment, with less variation between vaccination time points. In addition, the QuantiFERON SARS-CoV-2 assay does not differentiate between CD4^+^/CD8^+^ subsets because the epitopes are all mixed in one tube and only detect IFN-γ-secreting effector T-cells. Another limitation is that the T0, T1, and T2 parameters for IFN-γ secretion in response to COVID-19 antigens (Ag1, Ag2, and Ag3) were not normally distributed. Therefore, we were unable to correct for the confounding effect of different intervals between vaccine shots because the data violated the normality assumption and the study followed the repeated measures design with no predictor variable to predict the outcome.

In this study of recently immunized cancer patients, the BNT162b2 and the BBIBP-CorV vaccine induced significant neutralizing IgG antibody titer and a CD4^+^/CD8^+^ T-cell response against SARS-CoV-2. There was no significant difference between the first and second vaccine doses regarding T-cell response. Nevertheless, there was a significant difference between the first and second doses compared to the baseline naive state, indicating that the T-cell response is generated after the first vaccine dose and increases after the second dose, regardless of the vaccine type. Hence, it is recommended for cancer patients, as in healthy individuals, to receive both doses, as neither was associated with substantial adverse effects. In addition, cancer patients could produce IgG antibodies specific to SARS-CoV-2, which coincided with the COVID-19 T-cell response. This study is one of the first prospective cohort studies in cancer patients to quantify cellular and humoral SARS-CoV-2-specific responses at various time points before and after receiving COVID-19 vaccines. In addition, this is one of the first reports to compare multiple types of vaccines in cancer patients regarding cellular and humoral immune response; even fewer longitudinal studies have investigated efficacy after each time point. Furthermore, this study also provides valuable insights into the relationship between vaccine-induced T-cell responses and SARS-CoV-2-induced humoral immune responses in cancer patients on active cancer therapy.

Understanding the cellular and humoral immune response to COVID-19 in cancer patients undergoing active treatment will be critical in developing more effective vaccines and prevent future SARS pandemics. In addition, this informative study provides an example of evidence-based research that might be utilized to assure cancer and immunocompromised patients of the importance of receiving COVID-19 vaccines and developing vaccine protocols for them. It also highlights the significance of developing a T-cell-based vaccination capable of creating diverse memory B cells against SARS-CoV-2 in cancer patients. Overall, this research paper contributes to the ongoing efforts to understand the immune response to COVID-19 in cancer patients on active treatment and has important implications for clinical practice and future research.

## Figures and Tables

**Table 1:** Participating in cancer patients’ demographic information.

Variables		n (57)	%
Gender	Male	13	22.81
	Female	44	77.19
Age	median	51.27	
	Mean ± SD	49.9 ± 11.6	
Cancer category	Solid tumors	44	77.19
	Hematological malignancies	13	22.81
Type of vaccine	BNT162b2	30	52.63
	BBIBP-CorV	27	47.37

**Table 2:** The frequency of cancer patients receiving active therapy during sample collection.

Type of Active Cancer Therapy	n	%
Chemotherapy	23	40.4
Hormonal therapy	10	17.5
Radiotherapy	1	1.8
Immunotherapy	2	3.5
No therapy	21	36.8

**Table 3:** Correlation between IgG antibody titer (AU/mL) and IFN-γ secretion (IU/mL) in response to COVID-19 antigens at each time point after vaccination.

Antibody Titer (AU/mL)	Spearman’s Rho	Ag1—T1	Ag2—T1	Ag3—T1
T1	Correlation Coefficient (r_s_)	0.4	0.35	0.39
	*p*-value 2 tailed	0.005 *	0.016 *	0.008 *
		**Ag1—T2**	**Ag2—T2**	**Ag3—T2**
T2	Correlation Coefficient (r_s_)	0.4	0.41	0.33
	*p*-value 2 tailed	0.017 *	0.013 *	0.05 *

Antigen 1 (Ag1): SARS-CoV-2—CD4^+^ T-cell epitopes derived from the spike protein’s S1 subunit (receptor binding domain (RBD). Antigen 2 (Ag2): SARS-CoV-2—CD4^+^ and CD8^+^ T-cell epitopes from the S1 and S2 subunits of the S protein. Antigen 3 (Ag3): SARS-CoV-2—CD4^+^ and CD8^+^ epitopes from S1 and S2 plus immunodominant CD8^+^ epitopes derived from the SARS-CoV-2 genome. Time point 1 (T1): The blood sample was drawn 14–21 days after the first vaccination. Time point 2 (T2): The blood sample was drawn 14–21 days after the second vaccination. * By normal standards, the association between the two variables is considered statistically significant.

## Data Availability

Data available on request due to restrictions, e.g., privacy or ethical.
